# Confocal Scan Features of Keratic Precipitates in Granulomatous versus Nongranulomatous Uveitis

**Published:** 2011-10

**Authors:** Mozhgan Rezaei Kanavi, Masoud Soheilian

**Affiliations:** 1 Ophthalmic Research Center, Shahid Beheshti University of Medical Sciences, Tehran, Iran; 2 Central Eye Bank of Iran, Tehran, Iran

**Keywords:** Confocal Scan, Keratic Precipitate, Granulomatous Uveitis

## Abstract

**Purpose:**

To compare the morphologic features of keratic precipitates (KPs) by confocal microscopy in granulomatous versus nongranulomatous noninfectious uveitis.

**Methods:**

KP morphology was determined by confocal scan in patients with noninfectious granulomatous and noninfectious nongranulomatous uveitic cases.

**Results:**

One hundred and twenty-seven eyes of 90 subjects with noninfectious uveitis were studied. Thirty-nine eyes had granulomatous and 88 had nongranulomatous uveitis. Smooth-rounded KPs were significantly more common in the granulomatous subgroup (P<0.001) while cruciform and dendritiform KPs were more frequent in nongranulomatous uveitis (P<0.001 and P<0.005 respectively).

**Conclusion:**

Confocal scan may be used as an adjunctive tool for differentiating granulomatous from nongranulomatous uveitis. Smooth-rounded KPs are strongly suggestive of granulomatous inflammation.

## INTRODUCTION

In vivo confocal microscopy (IVCM) is a noninvasive technique that can demonstrate anatomic details of all organs at tissue level. It has been used successfully to visualize corneal lesions and other pathologies, such as loss of keratocytes and microscopic changes in the corneal epithelium or endothelium, which are generally not detected by other clinical or diagnostic methods.^1^ Keratic precipitates (KPs), are characteristic features of various types of intraocular inflammation. These are cellular aggregates on the corneal endothelium, typically composed of epithelioid cells, lymphocytes and polymorphonuclear cells.^2^ With the use of IVCM, Wertheim et al^3^ demonstrated six different types of KPs including globular, infiltrating, smooth-rounded, stippled, dendritiform and cruciform.

Kanavi et al^4^ evaluated confocal scan features of KPs in uveitic eyes of various etiologies and concluded that certain types of KPs were more frequently associated with specific forms of uveitis; smooth-rounded KPs might indicate granulomatous uveitis while infiltrating and dendritiform KPs could reflect infectious uveitis. Similar findings were reported by Wertheim et al^3^ and Mahendradas et al^5^. The current study was conducted to compare morphologic patterns of KPs evaluated by IVCM in granulomatous versus nongranulomatous noninfectious uveitis.

## METHODS

This observational comparative study was conducted from 2005 to 2010 and included patients with noninfectious uveitis who were further categorized as having granulomatous or nongranulomatous inflammation based on extensive clinical and laboratory evaluations. All patients underwent detailed ophthalmologic evaluation, including anterior and posterior segment evaluation together with review of systems by a uveitis specialist (MS). After obtaining approval from the Ethics Committee at the Ophthalmic Research Center, Shahid Beheshti University of Medical Sciences, patients were referred for IVCM (ConfoScan 3.4, Nidek technologies, Padova, Italy) of the involved eye(s) by an ophthalmic pathologist (MRK) to determine KP morphology.

After topical anesthesia with tetracaine 0.5% eye drops and placing methylcellulose as a coupling agent on the front lens (40×, 0.75 objective lens), corneal confocal scanning was performed. Both the automatic and manual modes were used to capture images of KPs starting from the center and proceeding toward the corneal periphery. The coronal section of each image was 455μm horizontally and 340μm vertically with lateral resolution of 1μm, and depth of field of 10μm. The six umbrella terms proposed by Wertheim et al^3^ were used to describe KP morphology. After specifying the morphologic features of KPs in each uveitic subgroup, data were analyzed using Fisher’s exact test and Chi-square tests. Predominant KP morphology was defined by the observation of at least 60% of a certain type of KP in each study subgroup.

## RESULTS

**Figure 1:**
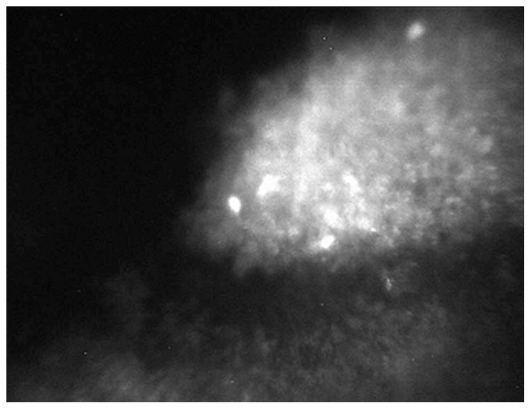
A hyper-reflective smooth rounded KP (magnification × 500).

**Figure 2:**
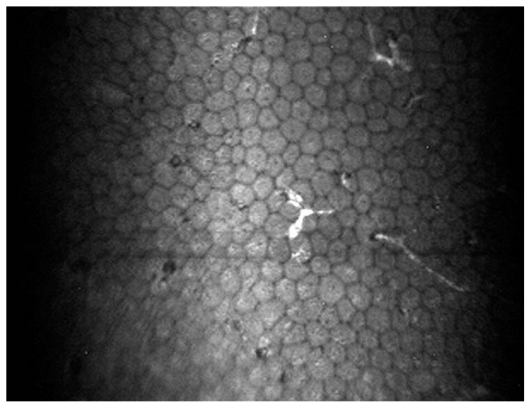
Few hyper-reflective cruciform KPs (magnification × 500).

**Figure 3:**
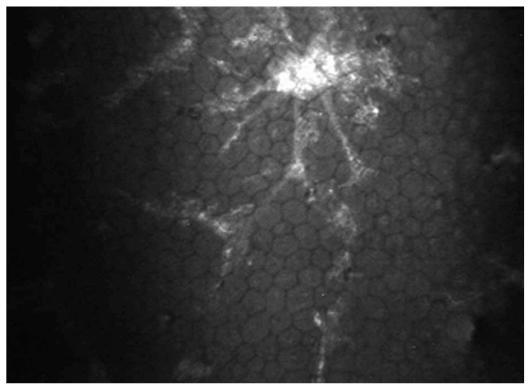
A typical example of high contrast dendritiform KP (magnification × 500).

**Figure 4:**
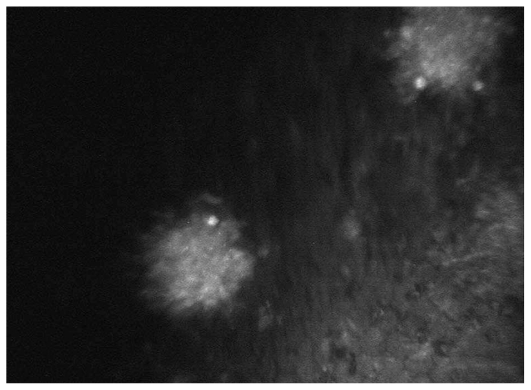
A couple of high contrast globular KPs (magnification × 500).

**Figure 5:**
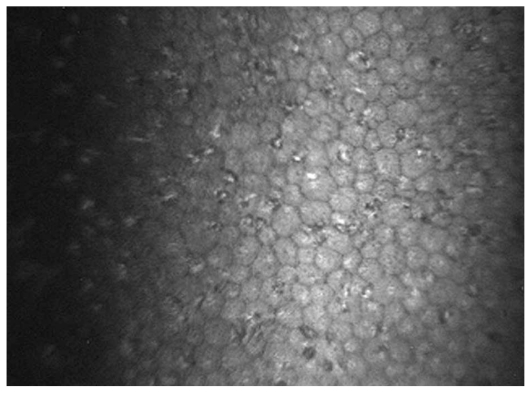
Diffuse hyper-reflective stippled KPs (magnification × 500).

**Figure 6:**
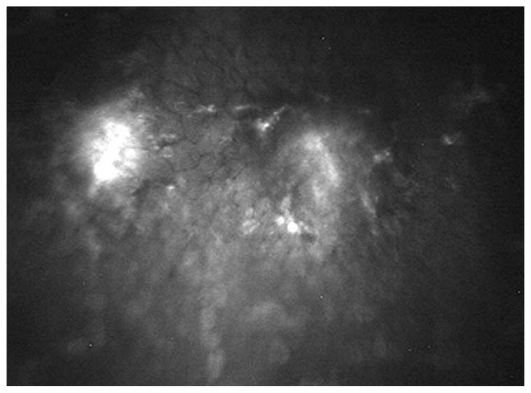
A couple of high contrast, infiltrating KPs (magnification × 500).

Overall, 127 eyes of 90 patients with non-infectious uveitis including 54 (60%) female subjects with mean age of 30.5±10.8 (range, 14–67) years were enrolled. Thirty-nine eyes of 23 patients had granulomatous uveitis including idiopathic granulomatous uveitis in 27, Vogt-Koyanagi-Harada syndrome in 8, sarcoidosis in 3 eyes, and sympathetic ophthalmia in one eye. Eighty-eight eyes of 67 patients had nongranulomatous uveitis including Fuchs heterochromic iridocyclitis (FHIC) in 63, pars planitis in 9, multiple sclerosis (MS) associated uveitis in 7, acute anterior uveitis in 3, sclerouveitis in 2, psoriasis-associated uveitis in 2, and HLA-B27 associated uveitis in 2 eyes. Smooth-rounded KPs (Fig. 1) were significantly more common in the granulomatous subgroup (P<0.001) whereas cruciform (Fig. 2) and dendritiform (Fig. 3) KPs were more frequent in the nongranulomatous subgroup (P<0.001 and P<0.005, respectively). Globular (Fig. 4), stippled (Fig. 5) and infiltrating (Fig. 6) KPs were common features in both granulomatous and nongranulomatous uveitis (P values, 0.2, 0.08 and 0.3, respectively).

## DISCUSSION

IVCM may be used for morphometric analysis of KPs in certain types of uveitis and facilitate the diagnosis and management of intraocular inflammation. Morphologic features of KPs in various types of uveitis have been evaluated with IVCM and specular microscopy in several studies.^3^–^9^ Our study demonstrated that the predominant KP morphology in granulomatous noninfectious uveitis is smooth-rounded, as compared to cruciform and dendritiform in nongranulomatous non-infectious uveitis. Smooth-rounded KPs have previously been reported in granulomatous uveitis.^3^,^6^ In one of these reports^3^, the appearance of KPs in 7 patients with idiopathic granulomatous uveitis was predominantly globular (71.4%) while smooth-rounded KPs were seen less frequently (28.6%). In another report^6^, predominant KP morphology in 7 eyes with idiopathic granulomatous uveitis was globular, smooth-rounded and stippled. In our study however, the prevalence of globular and stippled KPs was comparable in granulomatous and nongranulomatous uveitis.

Granulomatous inflammation is a proliferative inflammation characterized by infiltration of epithelioid cells otherwise known as tissue macrophages. Histopathologic changes in granulomatous inflammation result from inability of the macrophage to effectively eliminate the pathogen. Lymphokines from activated T cells induce monocytes and macrophages to become activated macrophages. With prolonged antigenic stimulation, activated macrophages may differentiate into epithelioid cells and then giant cells. The multinucleated giant cells may be derived from fusion of several epithelioid cells.^10^ The development of smooth-rounded KPs in granulomatous uveitis may be related to chronicity, activity of the inflammation and formation of multinucleated giant cells. Absence of such KPs in nongranulomatous uveitis may be explained by the lack of multinucleated giant cells in this type of inflammation. Further studies should be performed to correlate IVCM findings with histopathological features, i.e. a smooth-rounded KP may be a confocal scan feature of a multinucleated giant cell.

Cruciform KPs were previously reported in 19 of 40 eyes with FHIC.^7^ Such KPs have not been reported in other types of uveitis. In the current study, cruciform KPs were significantly frequent in eyes with FHIC but not so in other types of nongranulomatous uveitis. We occasionally observed cruciform KPs in a few cases of granulomatous uveitis. The frequent presence of such KPs in FHIC has not been accounted for yet. One should bear in mind that one morphologic type of KP may transform to another; this requires serial observations and IVCM examinations. In the current study, we performed confocal scan only once and as part of the diagnostic assessment. The major shortcoming of our study and that of the others^3^,^6^ was lack of frequent confocal microscopy to monitor changes over time; this should be the subject of future studies. The strength of our study was the relatively large number of cases with diverse etiologies, and verification of data using statistical and comparative analysis.

Although dendritiform KPs are strongly suggestive of infectious uveitis^3^–^5^, in the current study, they were observed in noninfectious uveitis including idiopathic granulomatous uveitis, pars planitis, MS-related uveitis, sarcoidosis, and acute anterior uveitis. To demonstrate an actual relationship between dendritiform KPs and infectious etiologies, further investigations including nucleic acid amplification tests of aqueous or vitreous specimens are necessary to validate the above-mentioned observations.

In Wertheim’s study, smooth-rounded KPs, in addition to granulomatous uveitis, were observed in 11.1% of eyes with infectious uveitis. Such KPs were not reported with infectious uveitis in Mahendradas’ study.^5^ We believe that infectious uveitic entities associated with granulomatous inflammation, such as herpetic uveitis, may be associated with smooth-rounded KPs.

In conclusion, IVCM plays a potentially important role in identification of underlying mechanisms in complex forms of uveitis and can be used as an adjunctive tool for differentiating granulomatous from nongranulomatous inflammation. Smooth-rounded KPs are strongly suggestive of granulomatous inflammation.
